# Supplementary material for: HIVprotI: an integrated web based platform for prediction and design of HIV proteins inhibitors
Source: J Cheminform. 2018 Mar 9;10:12. doi: 10.1186/s13321-018-0266-y (PMC5845081; doi:10.1186/s13321-018-0266-y)
Supplement: Supplementary file 2 — Additional file 2. Source code of HIVProtI web server. [file 13321_2018_266_MOESM2_ESM.zip › HIVprotI_Source-code/hivprotI/weka-3-6-6/doc/weka/core/xml/package-tree.html]

weka.core.xml Class Hierarchy


---


|  |  |  |
| --- | --- | --- |
| **Overview** \| **Package** \| Class \| **Tree** \| **Deprecated** \| **Index** \| **Help** \| **Weka's home** | |  |
| **PREV**   **NEXT** | **FRAMES**    **NO FRAMES**     **All Classes** |  |


---


## Hierarchy For Package weka.core.xml

**Package Hierarchies:**: All Packages

---

## Class Hierarchy

- java.lang.Object
  - weka.core.xml.**KOML** (implements weka.core.RevisionHandler)- weka.core.xml.**MethodHandler** (implements weka.core.RevisionHandler)- weka.core.xml.**PropertyHandler** (implements weka.core.RevisionHandler)- weka.core.xml.**SerialUIDChanger** (implements weka.core.RevisionHandler)- weka.core.xml.**XMLDocument** (implements weka.core.RevisionHandler)
            - weka.core.xml.**XMLInstances** (implements java.io.Serializable)
            - weka.core.xml.**XMLOptions** (implements weka.core.RevisionHandler)- weka.core.xml.**XMLSerialization** (implements weka.core.RevisionHandler)
                - weka.core.xml.**XMLBasicSerialization**
                - weka.core.xml.**XMLSerializationMethodHandler** (implements weka.core.RevisionHandler)- weka.core.xml.**XStream** (implements weka.core.RevisionHandler)

---


|  |  |  |
| --- | --- | --- |
| **Overview** \| **Package** \| Class \| **Tree** \| **Deprecated** \| **Index** \| **Help** \| **Weka's home** | |  |
| **PREV**   **NEXT** | **FRAMES**    **NO FRAMES**     **All Classes** |  |


---
